# Complement-targeted therapy: development of C5- and C5a-targeted inhibition

**DOI:** 10.1186/s41232-016-0013-6

**Published:** 2016-06-03

**Authors:** Takahiko Horiuchi, Hiroshi Tsukamoto

**Affiliations:** 1 Kyushu University Beppu Hospital, Beppu, Japan; 2 Kyushu University Graduate School of Medical Sciences, Fukuoka, Japan

**Keywords:** Complement, C5, C5a, C5a receptor, Membrane attack complex

## Abstract

The complement system is a major effector of humoral immunity and natural immunity. The complement system has three independent pathways of complement activation: a classical pathway, an alternative pathway, and a lectin pathway. These pathways converge to a common pathway that activates C3. This pathway also leads to the formation of various bioactive molecules such as C5a and the formation of membrane attack complex on the surface of target cells. In the past, the only preparations with anti-complementary action were C1 inhibitors (C1-INH), but an anti-C5 monoclonal antibody (eculizumab) appeared a few years ago, and this antibody has yielded encouraging results. In addition, a C5a receptor (C5aR) antagonist is in the clinical trial phase, and this antagonist should also prove efficacious. Anti-complement agents have garnered attention as a new treatment strategy for refractory inflammatory diseases.

## Background

The complement system consists of more than 30 proteins. These proteins are activated sequential order in a cascade that produces a variety of molecules that maintain homeostasis in the body by, for example, defending the body from infection. Complement activation results in the formation of membrane attack complex (MAC), and this complex produces holes in the cell membrane, causing the destruction of target cells. Complement fragments such as C3a, C4a, and C5a trigger inflammation as anaphylatoxins and chemotactic factors, and abnormal complement activation leads to various inflammatory diseases [1, 2].

Over the past few years, a monoclonal antibody against complement component C5 (eculizumab) has been approved as a treatment for paroxysmal nocturnal hemoglobinuria and atypical hemolytic uremic syndrome (aHUS), and this antibody has yielded encouraging results [3, 4]. Eculizumab binds to C5 and thus inhibits the cleavage of C5 into C5a and C5b, but its principal action is presumably by inhibiting the formation of C5b, which precludes the formation of MAC [5]. CCX168 was recently developed to inhibit inflammation caused by C5a. CCX168 is an orally administered C5a receptor antagonist. In a phase II clinical trial of CCX168 to treat anti-neutrophil cytoplasmic antibody (ANCA)-associated renal vasculitis (AARV), administration of CCX168 (+cyclophosphamide) was as efficacious as or more efficacious than the standard treatment (high-dose prednisolone + cyclophosphamide) [6, 7]. CCX168 has garnered attention as a potential replacement for corticosteroids, so CCX168 holds promise.

This paper outlines the mechanisms of complement activation. This paper then describes anti-complement agents that are being put to practical use. Last, this paper describes a C5a receptor antagonist that is in development.

## Mechanisms of complement activation

**Fig. 1:**
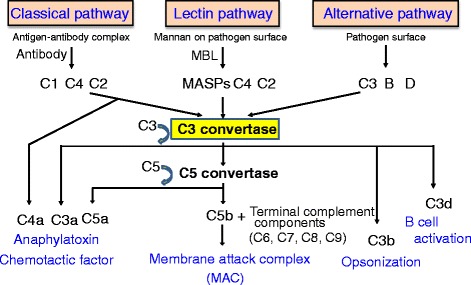
Complement activation. Three pathways, i.e., classical, lectin, and alternative pathways, are independently activated to form C3 convertase. The activation of this cascade culminates in the generation of various fragments derived from complement proteins and the formation of membrane attack complex (MAC). The former acts as potent mediators of inflammation by binding to their receptors on the cell surface, while the latter is comprised of C5b, C6, C7, C8, and multiple copies of C9 that generate lytic pores in cellular membranes

Complements are activated in three independent pathways: a classical pathway, an alternative pathway, and a lectin pathway (Fig. 1). These three pathways ultimately converge to a common pathway where they form a crucial enzyme known as C3 convertase. The classical pathway is triggered when the complement C1 complex binds to antibodies that are bound to antigens. Once C1 is activated, C4 and then C2 are activated. In the process, C4b and C2a are produced, and these two fragments combine to form C3 convertase. The lectin pathway is triggered when mannose-binding lectin (MBL) recognizes mannose or other carbohydrate sugar residues on the surface of pathogens. Once MBL-associated serine protease (MASP) is activated, C4 and then C2 are activated. Like in the classical pathway, C4b2a functions as C3 convertase in the lectin pathway. In the alternative pathway, complement factor D is directly activated by the surface of foreign particles. Once factor D is activated, factor B and then complement component C3 are activated. In the process, C3b and Bb are produced, and these two fragments combine to form C3bBb. C3bBb functions as C3 convertase on the surface of foreign substances [1, 2].

C3 is broken down into C3a and C3b by C3 convertase. C3b binds to C3 convertase to form C5 convertase, and C5 is broken down into C5a and C5b. C3a, C4a, and C5a function as potent mediators of inflammation, binding to receptors on the surface of lymphocytes, macrophages, and endothelial cells. Other products of the cleavage of these components have biological functions. C5b binds to the surface of foreign particles. C5b triggers the assembly of complement components C6 to C9 into MAC, which destroys foreign particles [1, 2].

Molecularly targeted therapies with antibodies are yielding dramatic results in the treatment of rheumatoid arthritis (RA) and malignancies. Complement activation by the antibodies themselves is crucial to their action [8, 9].

## Complement-targeting drugs that are being put to practical use

Complement activation serves to eliminate pathogens, but abnormal activation is heavily involved in the pathology of various conditions. As of today (February 2016), two drugs targeting specific complement components are being put to practical use. One is a drug that supplements a deficient complement while the other inhibits a complement.

1. C1 inhibitor (C1-INH) (Berinert®)

Hereditary angioedema (HAE) is due to a deficiency in the inhibitor of complement component C1 (C1-INH). A C1-INH has been refined from human plasma to supplement the lacking C1-INH. HAE is an autosomal dominant genetic condition in which an abnormality in the C1-INH gene causes a deficiency of C1-INH that in turn causes transient edema (angioedema) at various sites. The epidemiology of HAE in Japan is unclear, but studies in Europe and the USA estimate that HAE affects 1 in every 50,000 people. If acute edema appears in the eyelids and lips, it can also appear in the bowel and the larynx. Edema of the bowel causes intense abdominal pain, and edema of the larynx can cause asphyxia. Despite its rarity, these symptoms of HAE mean that it must be identified. During an episode of edema, administration of C1-INH can cause the episode to promptly end. Information on diagnosis and treatment of HAE can be found in the guidelines on hereditary angioedema from the Japanese Association for Complement Research [10].

2. Eculizumab, an anti-C5 monoclonal antibody (Soliris®)

Eculizumab is a humanized anti-C5 monoclonal antibody that binds to the α chain of C5. This prevents C5 from being cleaved into C5a and C5b by C5 convertase. As of February 2016, eculizumab is indicated for paroxysmal nocturnal hemoglobinuria (PNH) and atypical hemolytic uremic syndrome (aHUS), but a prospective clinical study is underway to expand its indications to include age-related macular degeneration, myasthenia gravis, optic neuritis, and preventing rejection of a kidney transplant [11].

PNH is a progressive hematologic disorder with a triad of features: complement-mediated intravascular hemolysis, bone marrow failure, and thrombosis. Japan is estimated to have a population of around 430 patients with PNH. The condition is caused by clonal expansion of hematopoietic stem cells with an acquired mutation in the PIG-A gene. In PNH, blood cells lack several types of proteins on their surface because the cells lack an anchor, glycosylphosphatidylinositol (GPI), to hold the proteins to that surface. These missing proteins include CD55 (a decay-accelerating factor, or DAF) and CD59 (a homologous restriction factor, or HRF) that would be present on the surface of various cells, such as red blood cells, to protect them from complement attack [12].

aHUS is defined as a condition with a triad of features: hemolytic anemia, thrombocytopenia, and acute renal insufficiency. The atypical form of aHUS is not associated with Shiga toxin, and the condition is not thrombotic thrombocytopenic purpura (TTP). aHUS has an extremely poor prognosis. aHUS due to genetic complement abnormalities can be treated with eculizumab. When surplus complement is activated in conjunction with abnormalities in various molecules involved in the alternative pathway of complement activation, vascular endothelial cells and platelets are activated and aHUS develops. Administration of eculizumab results in an improved prognosis for patients with aHUS [13]. A study has also suggested that eculizumab would be efficacious in treating HUS associated with Shiga toxin and TTP as well [14].

Eculizumab inhibits two complement functions, the anaphylatoxic action of complement component C5a and interaction with C5b that leads to the formation of MAC. Inhibition of the formation of MAC increases a patient’s susceptibility to a meningococcal infection, so caution is required. Thus, vaccination with a meningococcal vaccine prior to administration of eculizumab is recommended. Moreover, vaccination of small children with a pneumococcal vaccine and an influenza type B vaccine is also recommended.

## Complement C5a receptor antagonists

**Fig. 2:**
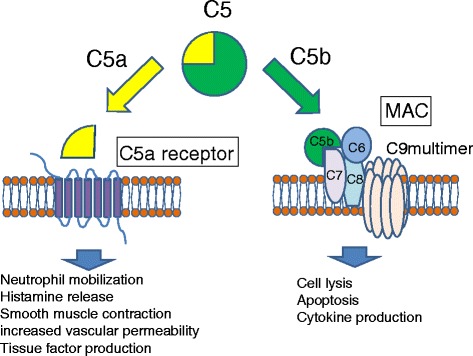
Activation of C5 by C5 convertase leads to the generation of C5a and C5b. C5a binds to C5a receptor and mediates various biological activities. C5b initiates the formation of MAC, which lyses cells as well as triggers inflammation

Attempts have long been made to develop inhibitors of complement C5 activation besides eculizumab [15]. These inhibitors target factors upstream of C5, including (in order) C5 convertase, complement components C5, C5a, and C5b, and C5a receptor (Fig. 2). Various types of antibodies and compounds such as peptides or non-peptides have actively been developed, and these substances act as inhibitors of complement components C5 and C5a and antagonists of the C5a receptor. These C5 and C5a receptor antagonists may be efficacious at treating various inflammatory diseases involving complements.

There is interest in the efficacy of these anti-complement agents on RA. In fact, numerous findings have suggested a relationship between RA and C5 and C5a receptors. C5a levels in synovial fluid are elevated in patients with RA [16], a genome-wide association study (GWAS) indicated that the TRAF1-C5 region is related to RA in humans [17, 18], numerous animal models have indicated that C5 is a gene responsible for causing arthritis [19, 20], and C5 and C5a receptor knockout mice are resistant to arthritis [21, 22]. However, clinical trials indicated that eculizumab (a humanized anti-C5 monoclonal antibody) and PMX53 and MP-435 (C5a receptor antagonists) had little efficacy in treating RA [23, 24].

Research has also suggested that complements are involved in causing ANCA-associated vasculitis (AAV) in humans. Patients with active AAV have significantly elevated C5a levels in plasma and urine in comparison to healthy people and patients with inactive AAV [25]. Bb levels in plasma are correlated with the disease activity of AAV in humans [26]. In a mouse model of nephritis induced with myeloperoxidase anti-neutrophil cytoplasmic antibody (MPO-ANCA), knocking out complement factor B and complement component C5 resulted in the absence of nephritis [27]. In light of these findings, researchers conceived of a model in which neutrophils stimulated by ANCA cause the release of substances that activate the alternative pathway of complement activation [27]. This activation results in the activation of complement factor B. Complement activation proceeds in association with the generation of C5a and MAC, producing nephritis. In fact, administration of an anti-C5 antibody in the mouse model of nephritis induced with MPO-ANCA almost entirely inhibited nephritis [28]. Knocking out the C5a receptor in the mouse model of nephritis induced with MPO-ANCA inhibited nephritis [29]. In addition, knocking in the human C5a receptor produced nephritis in mice [29]. When CCX168 (a C5a receptor antagonist) was administered in the mouse model of nephritis induced with MPO-ANCA, nephritis was inhibited in a dose-dependent manner [29]. Thus, the alternative pathway of complement activation is activated in MPO-ANCA-associated nephritis, and a C5a receptor antagonist may be efficacious in treating that nephritis.

The C5a receptor antagonist CCX168 was efficacious in mice, and the C5aR inhibitor on Leukocytes Exploratory ANCA-associated Renal Vasculitis (CLEAR) trial was conducted to determine if CCX168 would be similarly efficacious in humans [6, 7]. This trial was a randomized, double-blind, placebo-controlled phase II trial in which the C5a receptor antagonist CCX168 was orally administered to patients with ANCA-positive granulomatosis with polyangiitis (GPA), microscopic polyangiitis (MPA), or renal limited vasculitis. Patients were divided into three groups, with one receiving the standard treatment (cyclophosphamide 15 mg/kg IV pulses every 2–3 weeks + high-dose prednisolone 60 mg/day + a placebo; *n* = 9), one receiving CCX168 and prednisolone (cyclophosphamide 15 mg/kg IV pulses every 2–3 weeks + low-dose prednisolone 20 mg/day + 30 mg CCX168 twice daily; *n* = 8), and another receiving CCX168 but not prednisolone (cyclophosphamide 15 mg/kg IV pulses every 2–3 weeks + no prednisolone + 30 mg CCX168 twice daily; *n* = 8). The efficacy of each treatment was compared after 12 weeks. Administration of CCX168 was as efficacious as or more efficacious at inhibiting nephritis as the standard treatment. Moreover, CCX168 was efficacious in treating forms of disease activity besides nephritis. Patients receiving CCX168 were able to tolerate the drug, and they suffered no serious unanticipated adverse events. In addition to the CLEAR trial, the Clinical ANCA Vasculitis Safety and Efficacy Study of Inhibitor of C5aR (CLASSIC) trial is underway. This randomized, double-blind, placebo-controlled phase II trial intends to determine if CCX168 will allow a lower dose or discontinuation of corticosteroids for patients with AAV. Phase II pilot studies of CCX168 to treat aHUS and immunoglobulin A (IgA) nephropathy are also underway.

Eculizumab is an anti-C5 antibody, but C5a receptor antagonists target C5a alone and do not inhibit MAC, so those antagonists may lessen risk of an infection with a pathogen such as *Neisseria meningitidis* seen with eculizumab. However, caution is needed since the C5a receptor is involved in maintaining homeostasis in the body. In specific terms, the C5a receptor is expressed not only by hematopoietic cells but also by various non-immune cells, such as vascular endothelial cells, liver cells, kidney cells, lung cells, spleen cells, and cells of the gastrointestinal tract [30]. Long-term studies are needed to determine if blocking the C5a receptor has unanticipated effects.

## Conclusions

Research has revealed that several conditions respond to eculizumab and new attention has focused on eculizumab in relation to the treatment of conditions involving the complement system. This paper has described molecules functioning in the latter stages of complement activation, such as C5 and C5a receptors, but inhibitors of molecules functioning in the earlier stages of complement activation, such as complement factor D, complement factor B, properdin, MASP, and C3 convertase, are also being developed [11, 31]. These inhibitors may allow regulation of various steps in the complement system. Thus far, few attempts have been made to control disease by inhibiting complements, so those attempts hold promise as a new treatment strategy.
